# Development of a Simple Dipstick Assay for Operational Monitoring of DDT

**DOI:** 10.1371/journal.pntd.0004324

**Published:** 2016-01-13

**Authors:** Hanafy M. Ismail, Vijay Kumar, Rudra P. Singh, Christopher Williams, Pushkar Shivam, Ayan Ghosh, Rinki Deb, Geraldine M. Foster, Janet Hemingway, Michael Coleman, Marlize Coleman, Pradeep Das, Mark J. I. Paine

**Affiliations:** 1 Liverpool School of Tropical Medicine, Pembroke Place, Liverpool, United Kingdom; 2 Rajendra Memorial Research Institute of Medical Sciences (Indian Council of Medical Research), Agamkuan, Patna – 800 007, India; Universidad Autónoma de Yucatán, MEXICO

## Abstract

**Background:**

Indoor residual spraying (IRS) of DDT is used to control visceral leishmaniasis (VL) in India. However, the quality of spraying is severely compromised by a lack of affordable field assays to monitor target doses of insecticide. Our aim was to develop a simple DDT insecticide quantification kit (IQK) for monitoring DDT levels in an operational setting.

**Methodology/ principle findings:**

DDT quantification was based on the stoichiometric release of chloride from DDT by alkaline hydrolysis and detection of the released ion using Quantab chloride detection strips. The assay was specific for insecticidal *p*,*p`*-DDT (LoQ = 0.082 g/m^2^). Bostik discs were effective in post spray wall sampling, extracting 25–70% of active ingredient depending on surface. Residual DDT was sampled from walls in Bihar state in India using Bostik adhesive discs and DDT concentrations (g *p*,*p`*-DDT/m^2^) were determined using IQK and HPLC (n = 1964 field samples). Analysis of 161 Bostik samples (pooled sample pairs) by IQK and HPLC produced excellent correlation (R^2^ = 0.96; Bland-Altman bias = −0.0038). IQK analysis of the remaining field samples matched HPLC data in identifying households that had been under sprayed, in range or over sprayed.

**Interpretation:**

A simple dipstick assay has been developed for monitoring DDT spraying that gives comparable results to HPLC. By making laboratory-based analysis of DDT dosing accessible to field operatives, routine monitoring of DDT levels can be promoted in low- and middle- income countries to maximise the effectiveness of IRS.

## Author Summary

Visceral leishmaniasis (VL) is a major parasitic disease on the Indian Subcontinent, with 85% of the disease incidence in India. DDT is being used to control the sandfly vector of VL, but the quality of indoor residual spraying (IRS) is not routinely assessed due to a lack of practical assays. Here we have developed a simple DDT insecticide quantification kit (IQK) for measuring the amounts of DDT being applied to surfaces during large scale vector control operations. We demonstrate that the IQK provides equivalent data to the gold standard high performance liquid chromatography (HPLC) assay. Being truly point-of-care, this test is able to immediately inform the management of spray quality. Integration of the IQK will allow non-laboratory staff such as spray team supervisors to establish robust quality assurance procedures for DDT dosing that are vital to support the South Asian VL elimination efforts.

## Introduction

In India, DDT (2,2-bis(p-chlorophenyl)-1,1,1,-trichloroethane) is used extensively by the Indian National Vector Borne Disease Control Programme (NVBCDP) as part of efforts to eliminate VL [1]. VL is caused by *Leishmania donovani* and is transmitted by the female sand fly *Phlebotomus argentipes* [1]. An estimated 200 million people are at risk of this disease, of which 65 million live in India [2], predominantly in Bihar State [3,4]. When DDT is sprayed at a target dose of 1 g/ m^2^ for VL control, it can be efficient in reducing the indoor abundance of sand -fly populations [5,6]. However, scale-up of this program for national implementation resulted in insecticide levels significantly below the target dose [1,7,8], with up to 85% of walls being under sprayed in Bihar State [9].

Currently recommended approaches for quality assurance of IRS in vector control programmes falls into three categories: pre, during and post IRS quality assurance. Stock auditing, physically checking the state of spray equipment, servicing equipment at least once a year and rigorous training of IRS spray operators is done before IRS commences [10]. During operations supervision of spray operators by team leaders to ensure spray application technique is optimal and equipment malfunctioning is corrected, are advocated. In the case of DDT visual inspection of the powder delivered to the target surface is often used as an indicator of quality. Post IRS, WHO recommends cone bioassays to determine the quality of application of spray [10]. The imperative remains to develop practical field tools for close monitoring of DDT spray quality that allow reactive measures to poor spray quality.

The efficacy of IRS is maximized, when its coverage is sufficiently extensive and the correct concentration of active ingredient used to kill the insect population targeted. Under spraying with sub-lethal doses of insecticide reduces impact on the disease vector and facilitates evolution of insecticide resistance. Routine monitoring of insecticide residues reaching the surface is critical to ensure correct dosing but is not done as the tools available for estimating insecticide amounts on surfaces are not practical for field use. These include cone bioassays requiring live insects, which are non-quantitative and high performance liquid chromatography (HPLC), which is reliant on sophisticated and expensive systems, severely limiting their application in resource poor settings [11]. Most recently, colorimetric assays for cyanopyrethroids and carbamates have been developed for monitoring insecticide levels in bednets and sprayed structures [12–14]

DDT is an organochlorine molecule, thus quantifiable by enzymatic [15] or chemical [16] release of chloride ions (Cl^-^). Given the long development time and cost associated with producing enzyme based diagnostic assays and the fact that chloride detection kits such as Quantab Test Strips are available, we focused on developing a chemical platform for DDT detection with ‘off-the-shelf’ reagents that could be immediately deployed in the field. Here, we have developed a simple DDT dipstick assay and matched it against field samples taken in parallel with samples quantified by HPLC during a recent large-scale quality assurance survey of IRS operations in Bihar State, India in 2014 [9]. The results demonstrate that the IQK can provide accurate quantification of DDT, enabling the immediate implementation of robust quality assurance practices where IRS is used for vector control.

## Methods

### Chemicals and materials

Insecticide analytical standards *p*,*p`-*DDT (1.1.1-Trichloro-2.2-bis(*p*-chlorophenyl) ethane, Dichlorodiphenyltrichloroethane) 98.2% purity, *o*,*p’*-DDT 99.2% purity and DDE (1,1-dichloro-2,2-bis(4-chlorophenyl)ethylene) 99.5% were purchased from Chem Service (West Chester, UK). Potassium Hydroxide BioXtra, ≥85% KOH basis and Dicyclohexyl phthalate 99% purity were purchased from Sigma Aldrich (UK). HPLC grade solvents, heptane anhydrous 99% purity, 1-propanol anhydrous 99.7% purity, acetic acid 99.7% and HPLC grade water were purchased from Sigma Aldrich (UK). Low range Chloride Quantab Test Strips, 30–600 mg/L, were purchased from Hach (Hach Lange, Salford, UK).

### IQK assays

Step 1, DDT extraction: DDT samples (Bostik adhesive discs or filter) ~10 cm^2^ in area were cut into small pieces into a 50 ml Falcon tube. 1mL heptane was added and vortexed for 2–3 minutes. Step 2, Chloride release: 680 μl of the extract was transferred to a fresh tube, 100 μl of 2M KOH (dissolved in 1-propanol) was added, mixed vigorously for ~30 sec and incubated at room temperature (RT) for 1 hr. The reaction was neutralized with 100 μl of 2M acetic acid and the tube shaken vigorously for ~1 min then left to stand for ~1 min for phase separation. The bottom aqueous phase contains concentrated Cl^-^ ions. Step 3, DDT measurement: the bottom of Quantab strips were cut either side of the central wick to form a V shape and dropped into the tube with the wick immersed in the bottom aqueous phase. Strips were left for ~10 min for the Cl^-^ solution to reach the yellow indicator strip. Once the indicator turned blue, indicating saturation, the strips were removed and Cl^-^ readings in part per million (ppm) obtained by visual comparison of the chloride peak height against the manufacturer’s conversion chart. A *p*,*p’*-DDT quantitative chart in (g/m^2^) was also prepared for direct readout of DDT content as detailed below. To examine the effects of contaminating salts or alkaline compounds on Quantab readings, 0.01–1 mg of NaCl or Ca(OH)_2_ (lime) was added in Step1.

### Preparation of the *p*.*p’*-DDT conversion chart

Standard stock solutions of *p*,*p’*-DDT in the range 62.5–3000 μg/ml were prepared in 1 ml heptane. 680 μl of each standard were mixed with 100 μl of 2M KOH (1-propanol) and the IQK assay followed (Steps 2–3). Data was fitted to a third order polynomial (cubic)hyperbola curve using GraphPad Prism version 6.0 for Mac OS 10.10 (GraphPad Software, La Jolla California USA) and a DDT quantitative chart generated by interpolation of the manufacturers Quantab scale. The expected *p*,*p`-*DDT concentrations obtained from the curve were multiplied by a correction factor of 1.49 for heptane extraction efficiency (83.4%) and a third order polynomial (cubic) model underestimation value (80%) to give final readings of DDT in g/m^2^ resulted in DDT Quantitative chart to give a direct estimation of *p*,*p`*-DDT concentration in g/m^2^ *vs*. Quantab units on the scale.

### Assay performance

DDT breaks down into DDE and DDD. The linearity of DDE production and associated Cl^-^ release in the IQK assay was measured at RT and 40°C using DDT concentrations in the range 62.5 to 3000 μg/ml. The concentration of DDE was measured from the heptane phase following KOH treatment (IQK Step 2) by HPLC. 250μl of the heptane phase was evaporated to dryness under nitrogen and the residue dissolved in 1 ml methanol and centrifuged at 15000 rpm (Microcentrifuge Eppendorf; Model 5424R, UK). 10μl was injected into a reverse-phase Hypersil GOLD C18 column (175 Å, 250 x 4.6 mm, 5 μm, Thermo Scientific, UK) at 23–25^ο^ C. A mobile phase of acetonitrile/water 93:7 was used at a flow rate of 1 ml/min. DDT and DDE peaks were detected at 232 nm with an Ultimate 3000 UV detector (Dionex, Camberley, UK) and analysed by Dionex Chromeleon software. DDE and DDT sample concentrations were calculated from standard curves produced from known concentrations of DDE and *p*,*p’*-DDT standards. The HPLC method was linear in the range 0.064–400 μg/ml, with a mean coefficient correlation r^2^ ≥ 0.99998. The limits of detection and quantification were 0.008 and 0.032 μg/ml (RSD ≤ 2%).

### Post spray sampling

Adhesive (2x5) cm^2^ Bostik discs (Bostik, Leicester, UK) and Sellotape (10 cm^2^) were used. For comparison of extraction efficiencies, rough sided tiles were evenly coated by pipette with Indian DDT wettable powder formulation (DDT 50% WP) at variable concentrations up to 2g/m^2^. DDT recovered from the tile surface by adhesives were extracted by heptane and analysed by HPLC. Pearson’s correlation coefficient and Bland Altman analysis were used to assess systematic bias between the two analytical methods. To calculate sampling efficiencies of Bostik discs against different surfaces, ~0.25 m^2^ pieces of bamboo, thatch, brick un-plastered, brick plastered, brick lime washed and mud plaster surfaces were similarly coated with DDT formulation at 1 g/m^2^ of DDT 50% WP and left to dry for 1 day. IQK assays were performed in four replicates per surface, and the percentage *p*.*p’*-DDT recovered versus the quantity applied was calculated.

### Determination of DDT residues from field collected samples

Samples were collected from eight districts in Bihar State during the first DDT IRS round in 2014 (Feb–July) as described[8]. Briefly, two Bostik discs per HPLC sample (10 cm^2^ in total) and two adjacent discs for IQK were placed onto the wall surface and rubbed firmly. Bostik discs were removed from the wall surface using tweezers and placed on Whatman no. 1 filter paper, labelled and stored in a polythene bag at 4°C until analysis. Samples were taken from each of the four walls in bedrooms, at a different position per wall: high (4–6 feet from ground level), medium (2–4 feet from ground level), low (0–2 feet from ground level). The concentration of DDT present on wall surfaces was determined using HPLC[8] and IQK assay. A 20% cut-off threshold was used to classify results whereby a concentration of less than 0.8g/m^2^ was considered an under spray, a range of 0.8–1.2g/m^2^ was considered within the target range, and a concentration of greater than 1.2g/m^2^ was considered an overspray

### Determination of limits of blank, detection and quantification

Theoretical values for the limit of blank (LoB), limit of detection (LoD) and the limit of quantification (LoQ) were determined according to EP17 guideline [17].

### Statistical analysis

Statistical analyses were performed using GraphPad Prism version 6.0f for Mac OS 10.10, GraphPad Software, La Jolla California USA, www.graphpad.com and Microsoft Excel 2010. Correlation and Bland Altman statistics was used to compare and calculate the bias between analytical methods. Fisher’s exact test was used to evaluate statistical significance, with a cut-off *P* value of < 0.05. Generalized mixed-effects modelling using R v3.1.0 was used to analyse variance within individual households.

## Results

### DDT assay development

**Fig 1:**
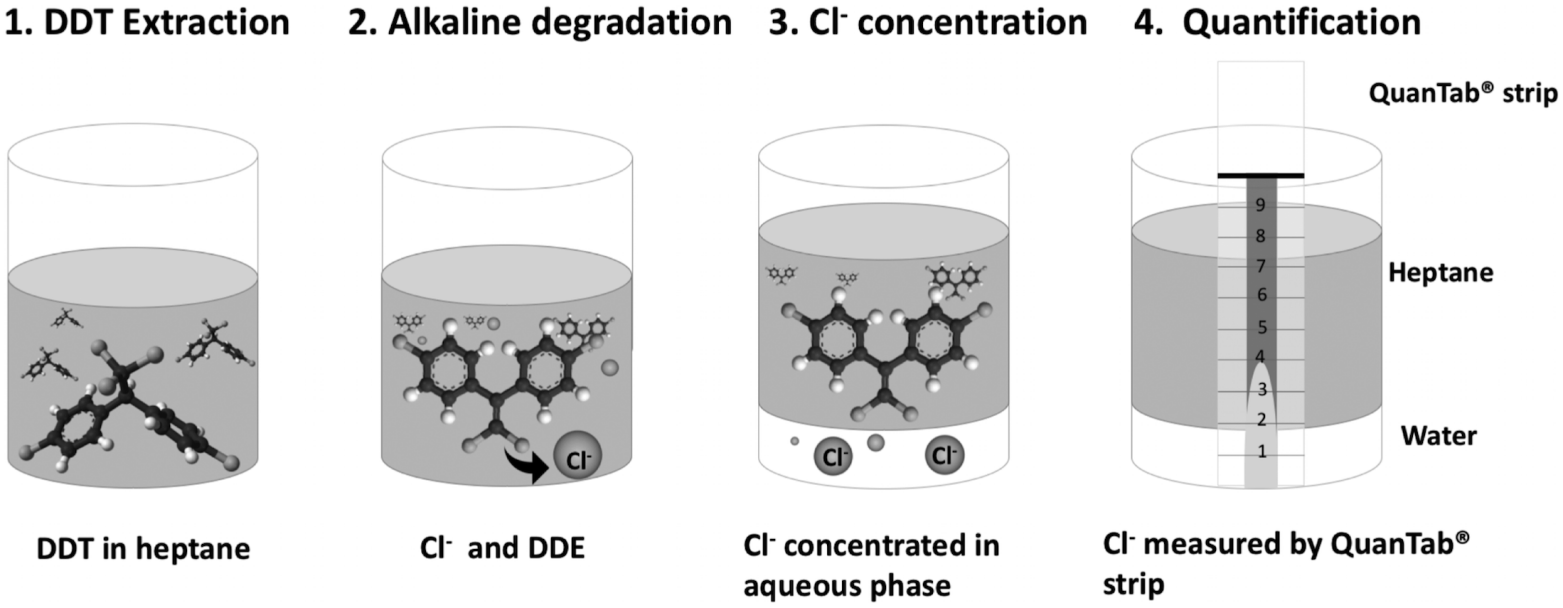
Schematic graph of the dipstick assay for determination of DDT. The assay operates through the concomitant release of chloride ion (Cl-) during alkaline-catalyzed DDT dehydrochlorination reaction that is measured by chloride Quantab Test Strips as follows: in Step 1 DDT is dissolved in heptane while contaminating ions remain undissolved and removed through transfer of DDT to a new tube. In Step 2 Cl^-^ is released by KOH, neutralized in Step 3 with ~1/10^th^ volume of aqueous acetic acid, which concentrates Cl^-^ into the bottom aqueous phase. In Step 4, Quantab strip measurements of Cl^-^ are taken from the aqueous phase allowing estimation of DDT concentration.

Alkaline treatment of *p*,*p’*-DDT results in stoichiometric Cl^-^ release (DDT: Cl^-^, w/w, ~10:1) which is easily measured using Chloride Quantab Test Strips that contain silver ions that form a visible white precipitate with chloride in sample solutions. The limits of Cl^-^ detection for the most sensitive strips are 30–600 ppm (S1 Fig), well below the solubility limit of DDT (0.025 ppm). To address this, heptane was used to solubilize DDT and a four-step process developed for Cl^-^ release, concentration and measurement (Fig 1). By dissolving DDT in heptane we were able to remove insoluble contaminant ions, which remain behind when DDT is transferred to a fresh tube. Similarly, by partitioning the Cl^-^ released by KOH into a small volume of aqueous acetic acid (stop solution), we were able to concentrate Cl^-^ into the measurable range of the Quantab strips.

**Fig 2:**
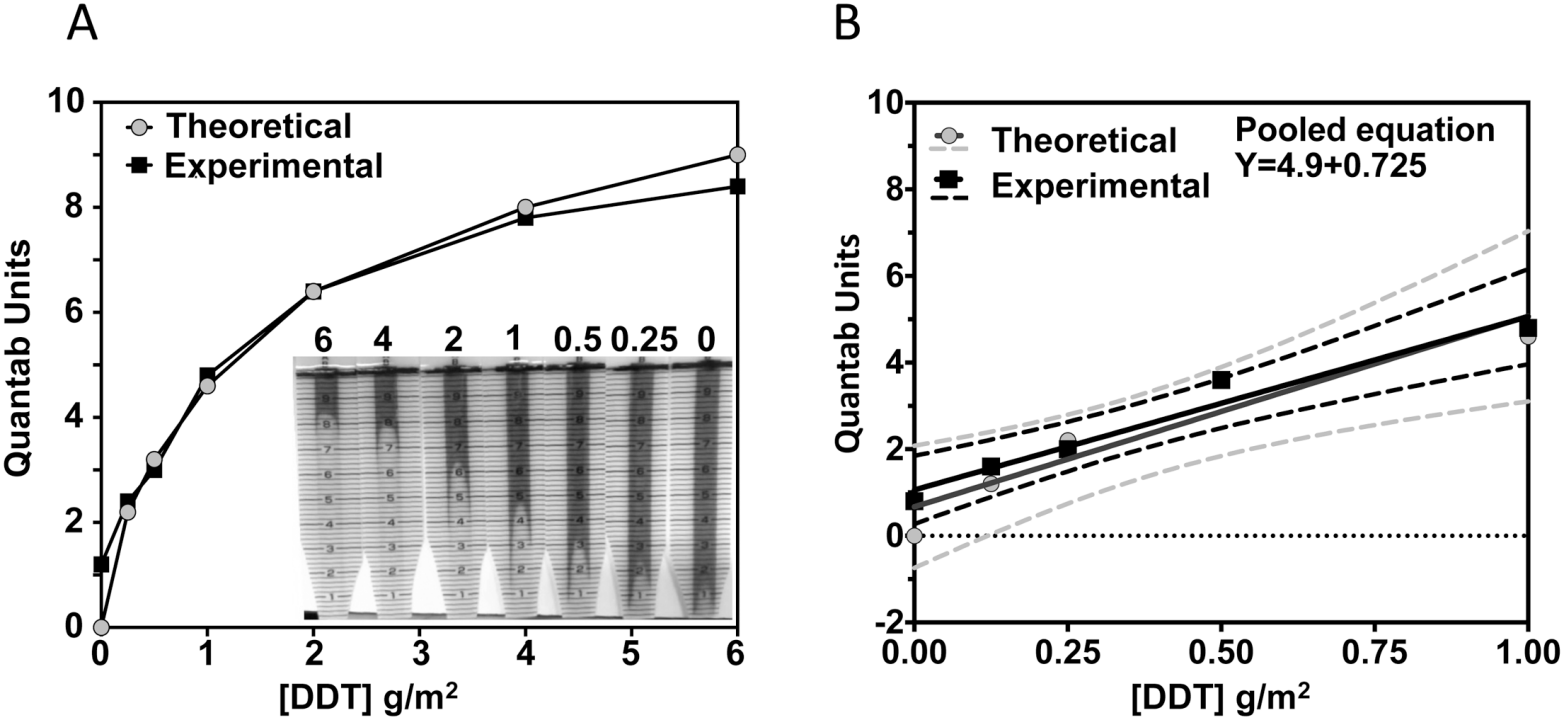
Comparison of theoretical chloride ion release from DDT and Quantab strip readings. A. hyperbolic range 0–6 g/m^2^. B. linear range 0–1 g/m^2^.

Quantab strip readings matched the theoretical (stoichiometric) calculations of chloride ions released from DDT, allowing simple estimation of DDT concentration (Fig 2). The limits of blank (unsprayed), detection and quantification were 0.009, 0.07 and 0.08 g/m^2^, respectively and the assay demonstrated high precision with RSD ≤ 4.4% at 1 g/m^2^ following IQK analysis of 24 sequential samples from rough tiles sprayed with 1 g DDT / m^2^ (S2 Fig). Equivalent DDT measurements were obtained at 20°C and 40°C (S3 Fig). DDT dehydrochlorination with concomitant release of DDE and Cl^-^ was linear in the DDT concentration range 0–4 mM, equivalent to 0 – 3g DDT/m^2^. There was 100% conversion of DDT to DDE with concomitant release of Cl^-^ as measured by Quantab strips within 60 min (S3 Fig). Overall, these data demonstrated that Quantab strips were able to measure DDT at the levels employed for IRS (1–2 g/m^2^) and under tropical temperatures (30–40°C)

The standard DDT WP formulation used for IRS contains a mixture of the active DDT ingredient *p*,*p`-*DDT (~80% of total DDT content), plus inactive DDT analogues including *o*,*p`-*DDT isomer (~15%) and DDE (~5%). Thus, the specificity of the assay was tested against non-target DDT analogues and biologically inactive DDT breakdown products often present in DDT formulations. There was no cross-reactivity with DDE or the inactive *o*,*p`-*DDT isomer at concentrations up to 2.8 mM (equivalent to 2 g/m^2^). In India, where this assay was field-tested, lime wash (Ca(OH)_2_) is commonly used to paint house walls, which could potentially cause false positive readings along with other residual ions. Neither sodium chloride nor lime effected Quantab readings (S4 Fig).

### Post spray sampling

**Fig 3:**
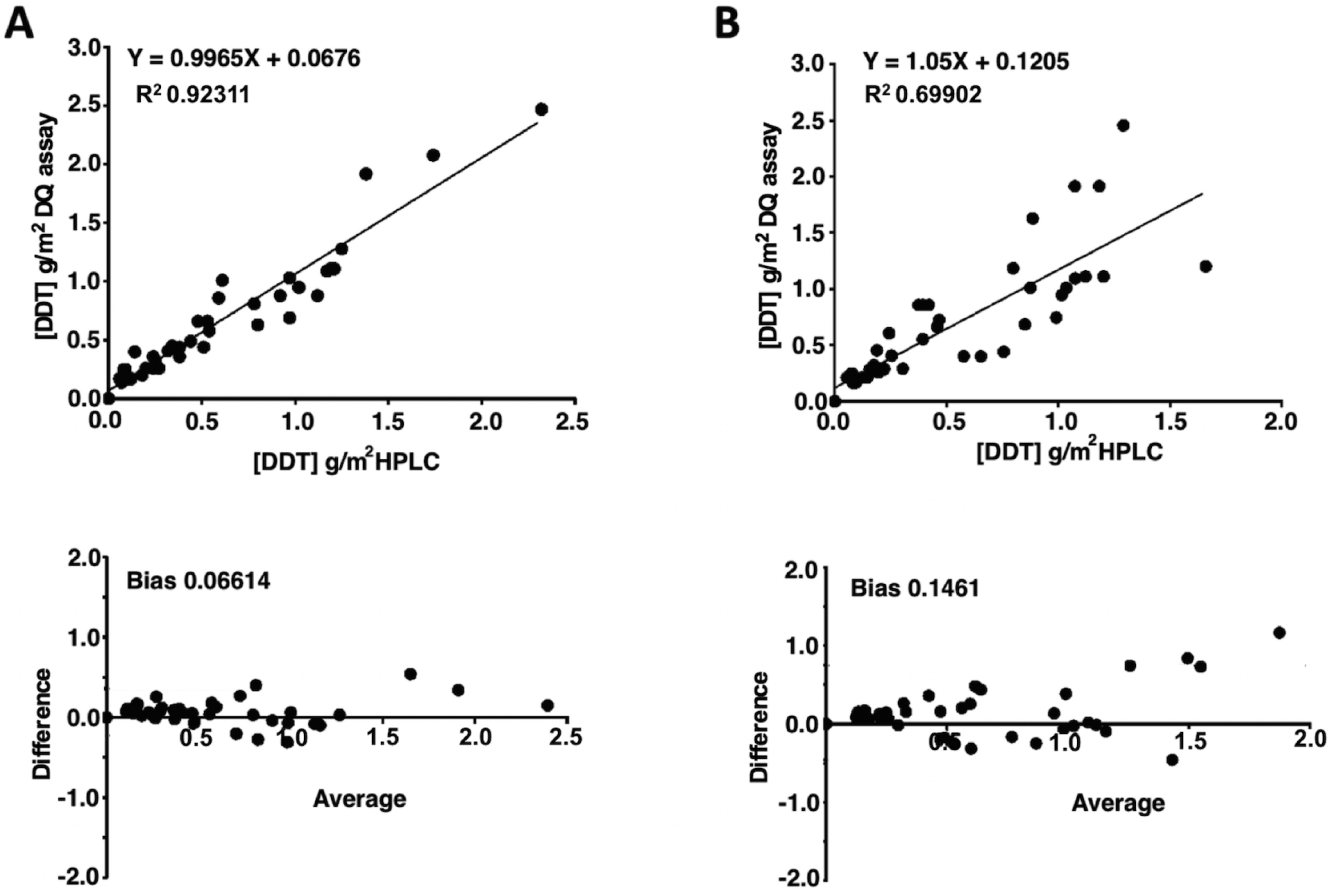
Comparison of DDT extraction from rough tile surfaces by Bostik adhesive discs and sellotape. A. Bostik disc correlation, R^2^ = 0.92311 (top), and Bland Altman bias, 0.06614 (below). B. Sellotape correlation, R^2^ = 0.69902 (top), and Bland Altman bias, 0.1461 (below).

**Fig 4:**
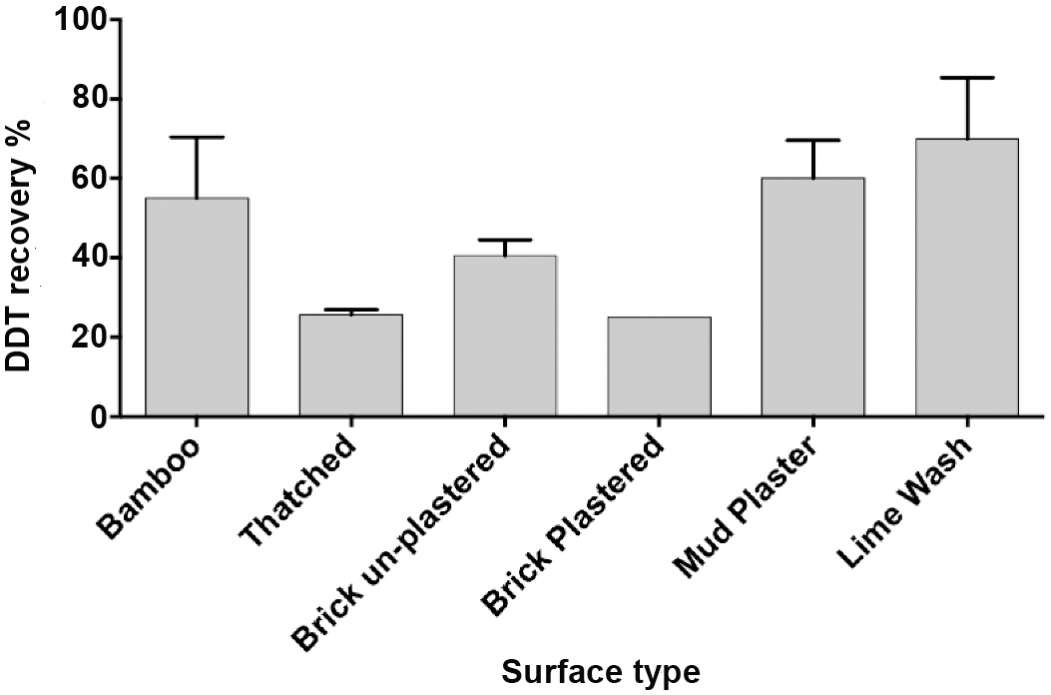
Comparison of DDT recovery by Bostik adhesive discs from different surface types. Error bars represent standard deviation from four independent replicates.

DDT is applied to surfaces as a wettable powder, thus tractable to post spray sampling using sticky tape removal of the surface spray layer [13]^,^[15]. Two different sampling methods were examined: normal adhesive tape (Sellotape) and Bostik adhesive discs. The rough side of tiles was used as an initial laboratory reference surface for estimating extraction efficiency. DDT was extracted from sprayed tiles and analyzed using IQK and HPLC (Fig 3). Bostik discs produced highly correlated IQK and HPLC measurements (R^2^ = 0.92; Bland Altman bias = 0.70) (Fig 3A) compared with Sellotape (R^2^ = 0.70; Bland Altman bias = 0.15) (Fig 3B), recommending Bostik discs for sampling. The average percentage recovery of DDT from rough tiles using Bostik adhesive discs was 53% +/- 5.6. DDT recovery was further examined using six typical Indian household surfaces (Fig 4). Recovery rates ranged between 25–70% depending on surface material and roughness (Bamboo 55.1%, Thatched 25.7%, Brick un-Plastered 40.5%, Brick Plastered 25%, Mud Plaster 60% and Lime Wash 69.9%), and these values were used for field estimations of DDT application.

### Determination of DDT from sprayed houses

In order to test the IQK as a method for capturing QA performance data, the IQK assay was matched against HPLC using 1964 Bostik disc samples (491 households) in Bihar state during the April–June 2014 DDT IRS season[8]. Double the numbers of samples were collected from each household in the post-IRS QA survey, and two types of analysis to test the IQK were performed. In the first, both pairs of Bostik discs underwent a common extraction process to obtain a uniform sample, which was then used for both HPLC and IQK (the ‘pooled testing’). In the second, each Bostik adhesive disc pair was analyzed independently (‘non-pooled testing’).

**Fig 5:**
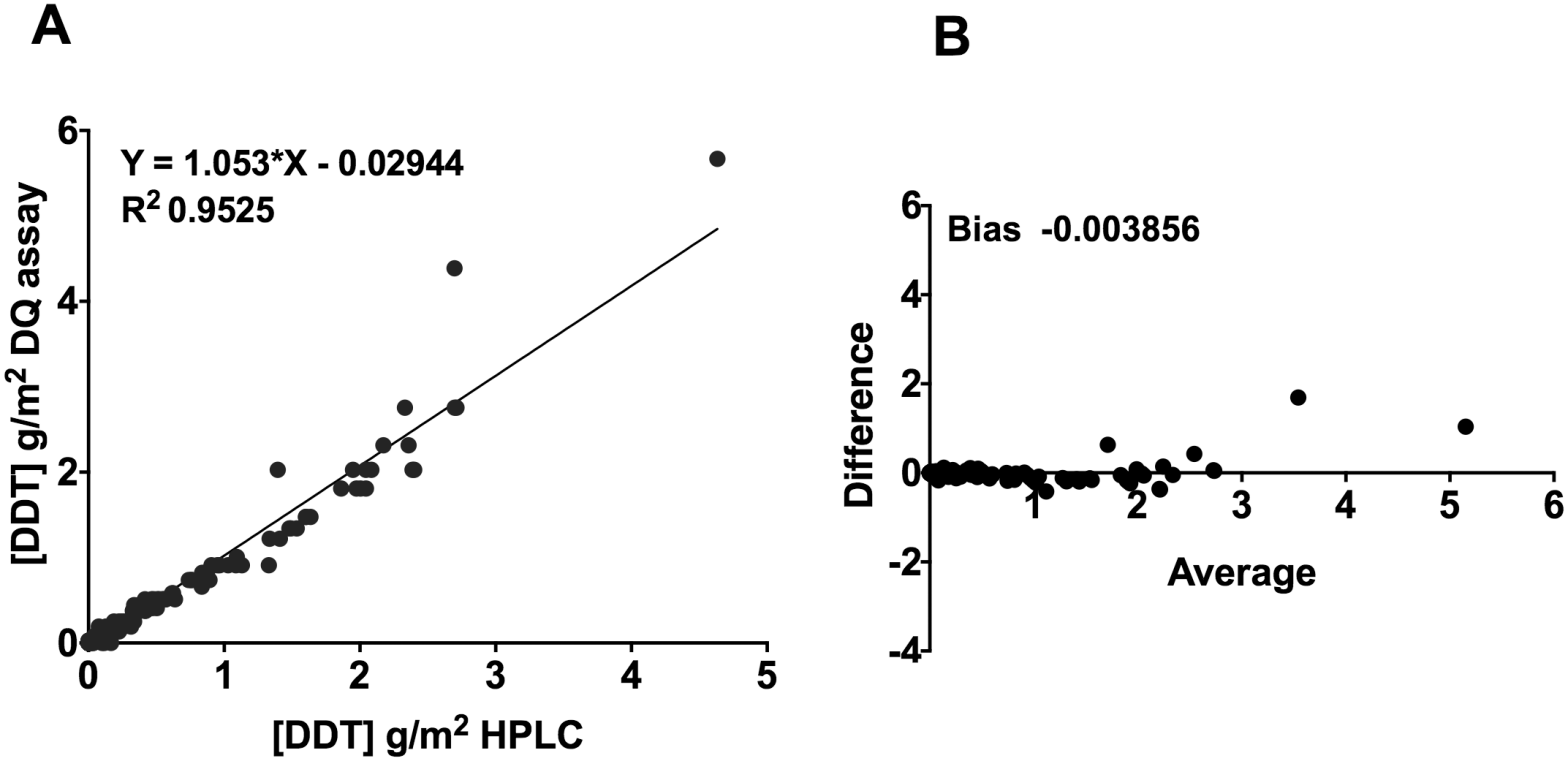
Comparison of DDT quantification of field samples (pooled). A. Bostik sample correlation of IQK vs HPLC; R^2^ = 0.9525, and B. Bland Altman bias, -0.003856.

One hundred and sixty one samples were tested using pooled analysis (Fig 5). The two assays showed an excellent level of correlation, with an R^2^ value of 0.96. The comparison of the IQK to HPLC using Bland-Altman analysis showed a very low bias of—0.0038, which confirmed that testing Bostik discs using the IQK was comparable with using HPLC.

**Fig 6:**
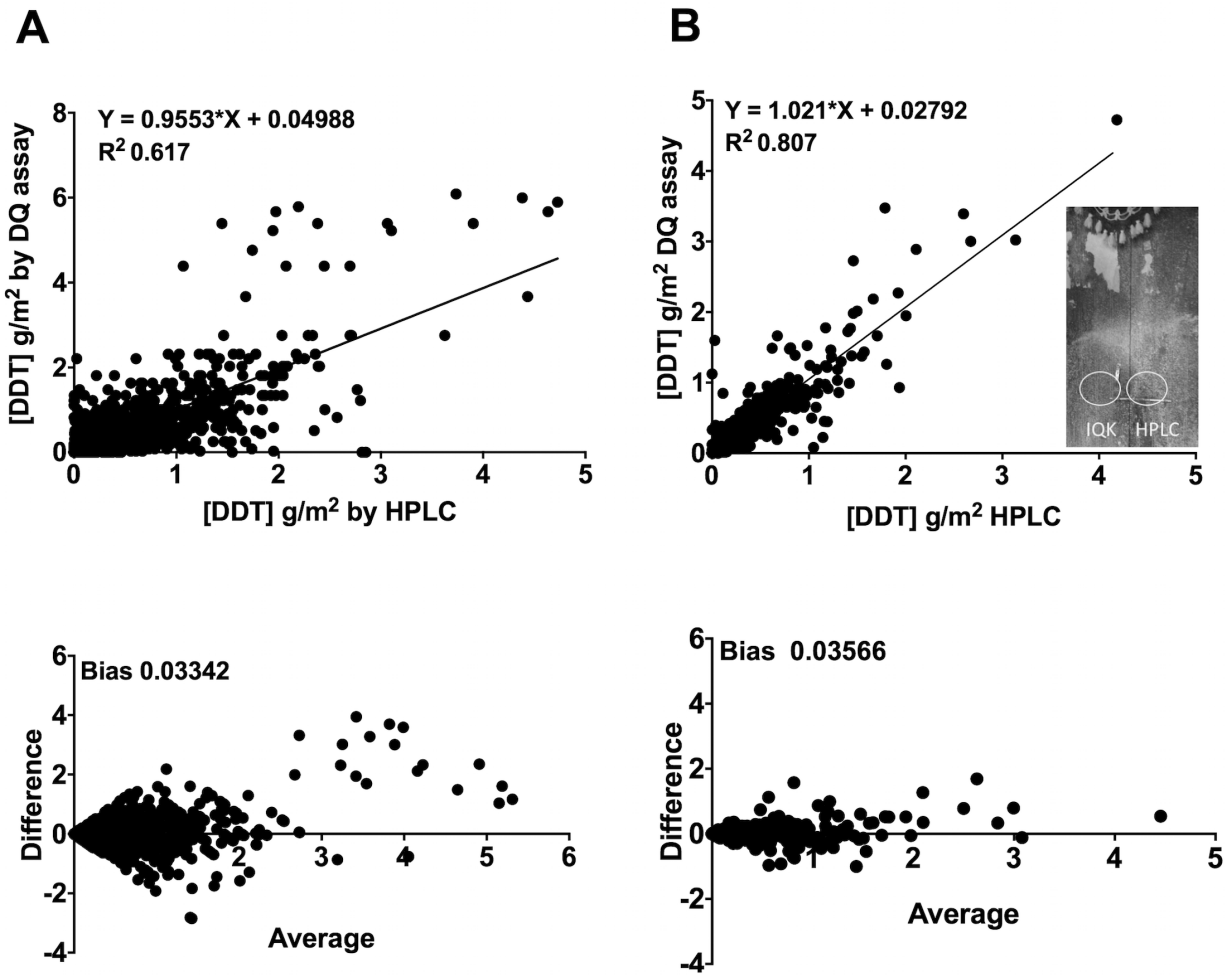
Comparison of DDT quantification of field samples by IQK and HPLC (non pooled). A. correlation of individual sample pairs (n = 1964 samples), R^2^ 0.617 (top), and Bland Altman bias, 0.03342 (bottom); B. correlation of average household values for sample pairs (n = 491 samples), R^2^ 0.617 (top), and Bland Altman bias, 0.03566 (bottom). Inset picture shows typical white DDT powder residue with IQK and HPLC sample areas circled.

The correlation of IQK versus HPLC for non-pooled sample analysis (1964 samples) was lower (R^2^ = 0.61) (Fig 6A), reflecting the inherent variation in the DDT wettable powder formulation delivered to walls; however, the Bland-Altman analysis for the non-pooled samples still showed a low bias of 0.01. Comparison of household averages increased correlation (R^2^ = 0.81; Bland-Altman bias = 0.03566) (Fig 6B) between the two methods. No significant interaction was found between the height of sampling and wall surface type (*P* = 0.7), and no significant effect of time of DDT sampling up to 15 days’ post DDT spraying was found (*P* = 0.5092, interaction *P* = 0.7331). The simple finger rubbing extraction procedure for recovering DDT from surfaces was consistent as measured by Levey-Jennings analysis of households sprayed within the correct target range (0.8–1.2g/m^2^ DDT); 94% were within 2SD of the mean DDT concentration (0.93 g/m^2^) (S5 Fig). The inclusion of data beyond 15 days of post spray sampling did show a significant effect on the concentration of DDT recovered from walls (*P* = 0.0031), although we believe this may be affected by the low number and uneven distribution of wall surface types in the post 15 days’ spray range.

**Fig 7:**
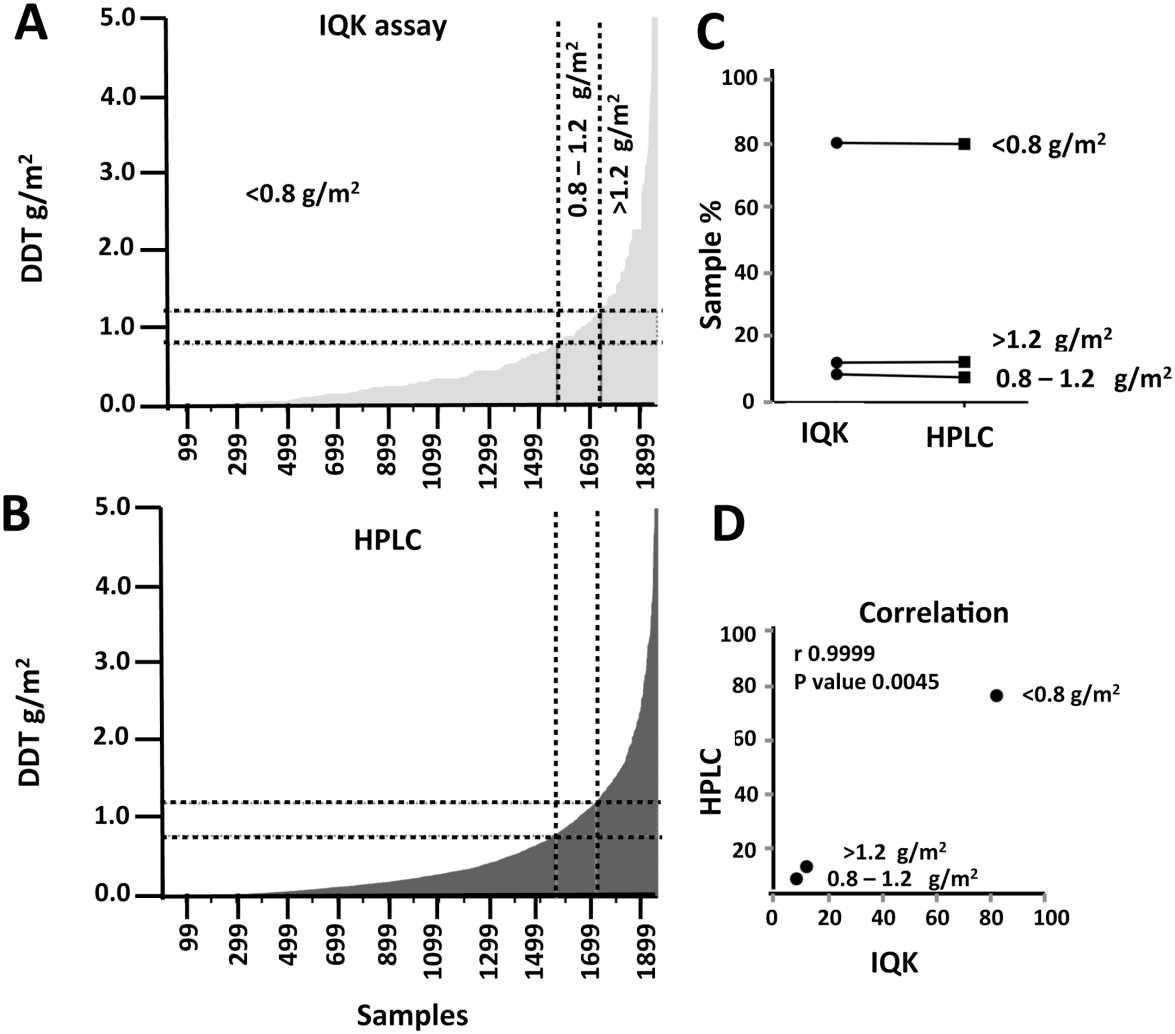
Comparison of DDT field sample measurements by IQK and HPLC. A. field collected samples analyzed by DQK assay (n = 1964). B. Field collected samples analyzed by HPLC (n = 1964). All samples corrected for DDT sampling efficiency of relevant Indian surfaces depicted in Fig 4 and further corrected for 71.5% *p*,*p`-*DDT content in Indian commercial formulation (50% WP-DDT). Data categorised according to cut-off values for low spray (<0.8 g/m2), target (0.8–1.2 g/m2) and overspray (>1·2 g/m2). C. Paired t-test comparison indicating no significant difference between HPLC *vs*. DQK cut-off (*P*-value = 0.4173) and D. Strong correlation between DQK and HPLC over the three levels with r = 0.9999 and *P* value = 0.0045.

**Fig 8:**
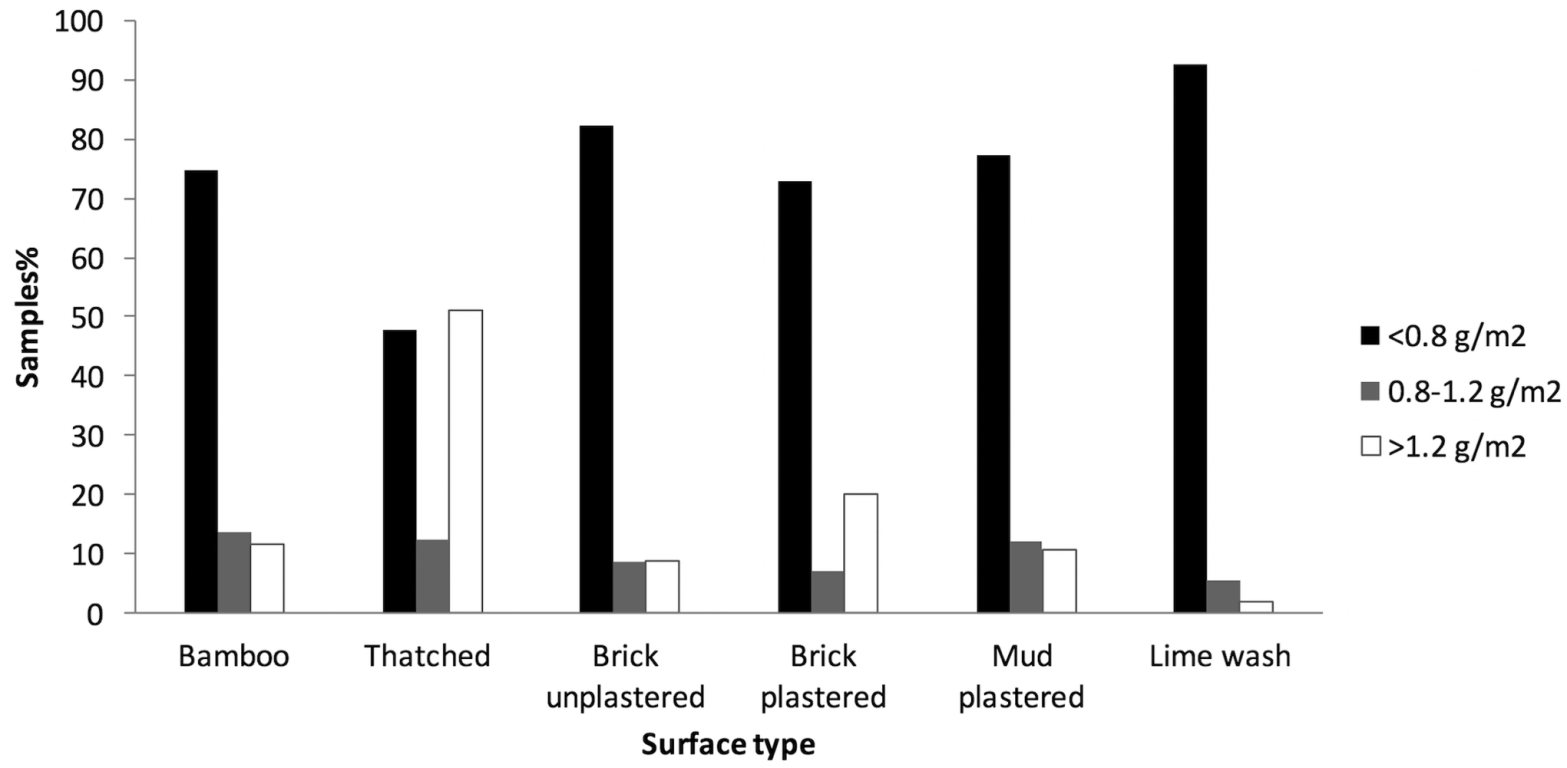
Spray profile of samples analyzed by IQK categorized by surface type.

Graphs comparing IQK and HPLC data from all the replicates from each sampled house are presented in Fig 7. For programmatic decision-making, cut-offs have been applied to indicate under spraying (<0.8 g/m^2^), correct spraying (0.8–1.2 g/m^2^), and over spraying (>1.2 g/m^2^)). There was no significant difference between the two methods (*P* = 0.4) (Fig 7B), and strong correlation in interpretation of the three dosage strata (r = 1.0, *P* = 0.005) (Fig 7C). Overall, poor quality spraying was evident with only 8.6% (*n* = 170) of samples at the target dose (0.8–1.2 g DDT/m^2^). Of the remaining samples, 11.2% (*n* = 220) were overdosed, and 80.1% (*n* = 1573) under-dosed. Similar levels of poor spray quality were evident for all surfaces apart from thatch (Fig 8), where a high level of over spraying (47%, n = 41) was evident.

## Discussion

At present, the operational response to poor quality spraying is severely limited by the cost and lengthy turnaround times for laboratory analysis of field samples. Although a range of new field tests for insecticide quantification are under development, including biosensors for DDT and pyrethroid detection [15,18], and colorimetric tests for cyano-pyrethroids [13,14], none are in routine use. By using ‘off-the-shelf’ reagents we have produced a simple low cost insecticide quantification kit for the detection of DDT that performs favourably against HPLC. As well as being expensive at ~£3.0 per assay excluding equipment cost, performing HPLC assays cannot be done on-site in the field, limiting programmatic ability to respond to QA failures. By comparison, material and reagent cost per test of our IQK assay is ~ £1.0, with quantitative results that are easily interpretable by untrained users within ~1.5 hours. In this study ~2000 samples were analysed at a rate of ~150–170 samples/ person/ day. The lower capacity and sample run times (20 minutes) of HPLC, in contrast, limited the number of samples analysable per day to ~100. The development of a simple field assay for measuring the active ingredient of DDT (*p*,*p’-*DDT) thus presents new capabilities for users to perform routine on-the-spot tests to ensure correct DDT dosing.

Quantification is achieved by measuring the Cl^-^ released following alkaline hydrolysis of DDT. The Cl^-^ released can be simply measured using Quantab strips and correlates with the initial DDT concentration in the range 0.04–3 mg/ml, equivalent to DDT formulation spray rates of 0.08–6 g/m^2^, covering the normal target range of 1–2 g/ m^2^ used for VL and malaria control operations respectively. The assay is highly specific for the biologically active *p*,*p’-*DDT molecule against the DDT breakdown products, while simple phased separation of DDT into heptane followed by separation of the Cl^-^ released into aqueous acetic acid alleviates interference by dirt or chemicals. Overall, the assay is far more practical compared with standard HPLC methodology, while the high specificity for *p*.*p’*-DDT provides an advantage over alternative immunoassay-based systems[18]. The assay is also adaptable to use with other chloride detection systems. For example, once Cl^-^ is released into the aqueous phase (Fig 1) AQUANAL -professional Chloride reagent test (FLUKA-UK) can be used alone or following Quantab strip measurements to produce a colorimetric reading for the Cl^-^ detection step.

The main obstacle in adapting the IQK for monitoring IRS is to develop a method to accurately sample insecticide from the walls of sprayed houses. Attaching large filter papers to walls prior to spraying [19], as recommended by WHO, creates behavioural bias due to the visibility of the papers, although this may be overcome using more discrete attachments such as small filter papers or felt pads [13]. Whilst the IQK can easily be used with pre-spray attachments, anonymous direct sampling of residues off wall surfaces is far more desirable. Since DDT is applied to walls as a wettable powder, Bostik adhesive discs were found to be a reliable method for extracting DDT for post spray measurement. The efficiency of DDT recovery ranged from 25 to 70% depending on surface type, which can be factored into the estimations of DDT application.

Water dispensable powders such as DDT have the advantage of low cost and long residuality on porous surfaces [20]. However, this is offset by rapid sedimentation of active ingredient and spray nozzle clogging that can produce non-uniform spraying, potentially impacting spray quality, and reinforcing the need for close monitoring of DDT spray quality. In order to assess the performance of the DDT IQK in measuring DDT, paired samples were taken from Indian households and either pooled (160 samples) for HPLC and DDT IQK analysis or analysed separately (1964 samples). The results for DDT IQK closely matched those of HPLC and were unaffected by surface contaminants collected from diverse surfaces (brick walls, wood, thatch and lime painted surfaces). Since DDT is applied in swathes using stirrup-pumps in India, the patchiness of the powder residues deposited on the walls was reflected by the different correlation coefficients of pooled vs non pooled paired samples for the HPLC vs IQK analysis (R^2^ = 0.96 *vs*. 0.64, respectively). However, the correlation of non pooled samples was strengthened (R^2^ = 0.8) when averaged household concentrations were compared. Overall, these data demonstrate the dipstick assay to be a robust method for quantifying DDT spray rates.

In this study, the IQK data confirmed that the rate of DDT spraying in Bihar State was consistently below the 1.0 g ai/m^2^ target concentration, with little variation in DDT concentrations within households or across districts [9]. Reasons for the reduced quality of the IRS have yet to be established, but include sub-standard spraying or sub-standard WHO specification formulation, both of which are measurable by IQK. The ability of the IQK to quantify the DDT active ingredient demonstrates that a simple dipstick assay can be used to verify that sprayers have actually sprayed a house, to evaluate the spray coverage in each house and to verify the effective insecticide coverage rates per area. Since DDT is applied at high concentrations (1–2 g/ m^2^) as a wettable powder the sticky adhesives work well in recovering the formulation after spraying. However, post spray recovery with adhesives may not be as efficient with microencapsulated formulations or low concentrations of active ingredient (mg range). As a rough estimate we have found the Bostik Discs pulls 40–50% of DDT off surfaces, but for accurate estimations, one must consider differences in surface recovery depending on the types of walls being sprayed. Here, DDT recoveries from common wall surfaces in Bihar have been factored into DDT estimations, but these might vary in different countries or states.

The availability of the simple dipstick assay for DDT quantification is an important step forward in enabling programme managers to report on the quality of their operations. From an operational perspective, the simplicity of the IQK means that large numbers of samples can be processed on a daily basis on-site. The next important step will be to develop standard operating procedures to information on spray quality that is relevant to spray operatives that will allow them to take appropriate steps in the case of spray failures

## Supporting Information

S1 Fig Calibration curve for Quantab low range (30–600 ppm) Cl^-^ quantification strip. A, hyperbolic range of Cl^-^ concentration and Quantab readings; (B) linear correlation range of the chloride calibration curve, R^2^ = 0.99197.
(TIFF)

S2 Fig Precision of the IQK assay. Levy-Jenning's analysis of 24 samples of DDT (1 g/m^2^) assayed by IQK. (TIFF)

S3 Fig Effect of temperature on alkaline mediated DDT dehydrochlorination. A, DDE production (HPLC measurement), B, Cl^-^ production (Quantab measurement), inset is correlation analysis for the linear portion of the DDT standard curve (0–1 g/m^2^) the top X-axis shows DDT rates (g DDT/m^2^) equivalent to the bottom X-axis DDT concentrations (mM). Reactions were carried out at 25°C (RT) and 40°, with 45 and 60 minutes incubation times.
(TIFF)

S4 Fig Effect of chloride salt and lime (Ca(OH)_2_) on Quantab readings. (TIFF)

S5 Fig Levy-Jenning's analysis of IQK data for households (n = 36) sprayed within the DDT target range for IRS (0.8–1.2 g/m^2^). (TIFF)
