# Supplementary figures and images for: Development of a Simple Dipstick Assay for Operational Monitoring of DDT
Source: PLoS Negl Trop Dis. 2016 Jan 13;10(1):e0004324. doi: 10.1371/journal.pntd.0004324 (PMC4711890; doi:10.1371/journal.pntd.0004324)

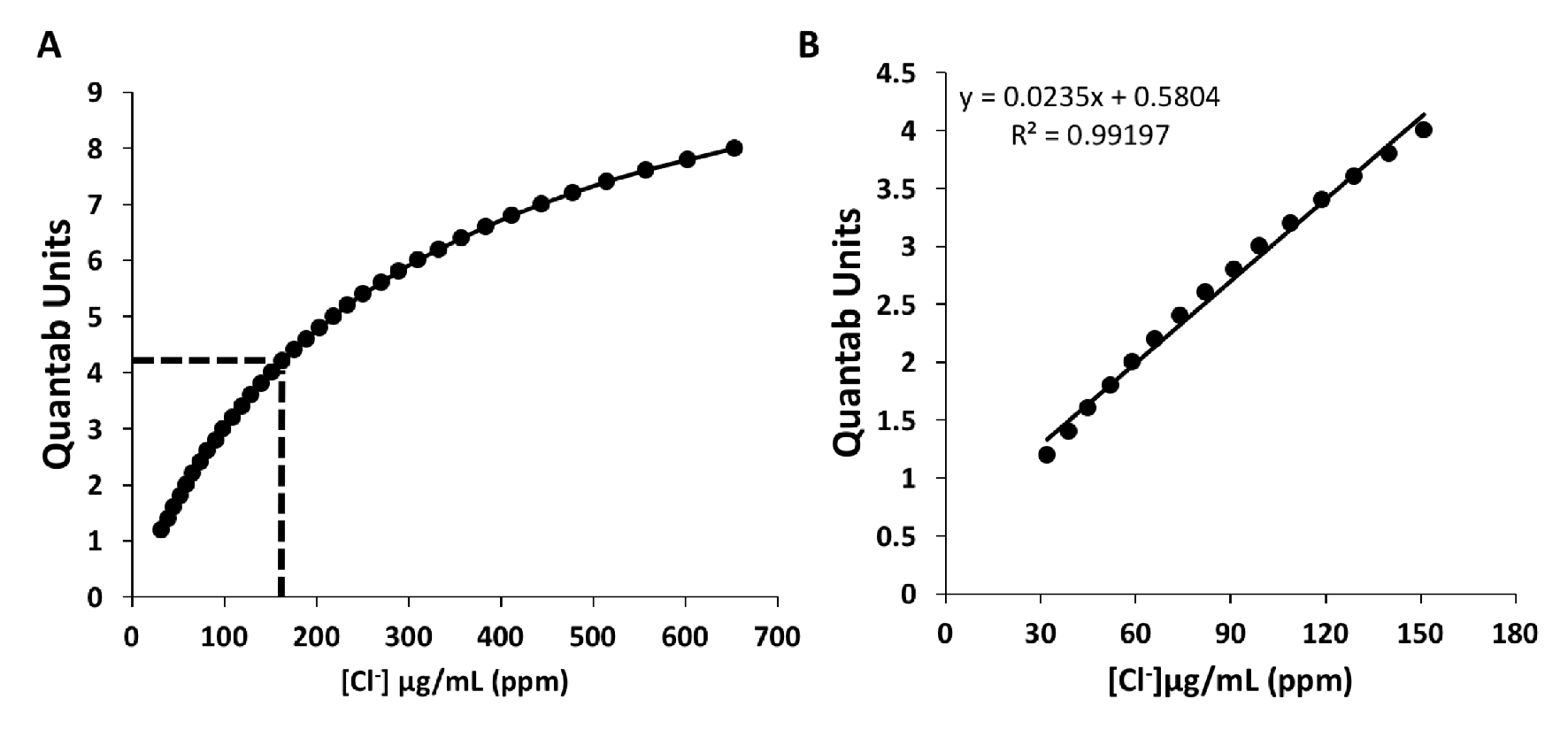

Supplement: S1 Fig — A, hyperbolic range of Cl- concentration and Quantab readings; (B) linear correlation range of the chloride calibration curve, R2 = 0.99197. (TIFF) [file pntd.0004324.s001.tiff]

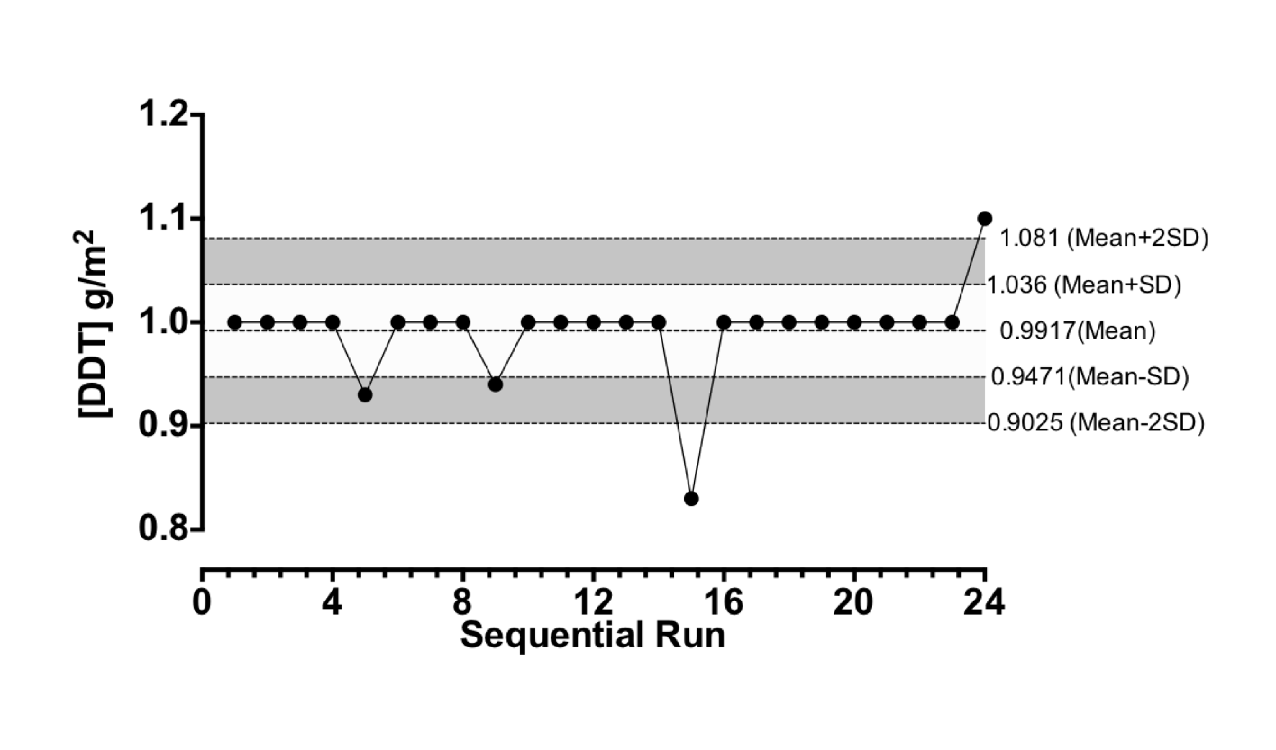

Supplement: S2 Fig — (TIFF) [file pntd.0004324.s002.tiff]

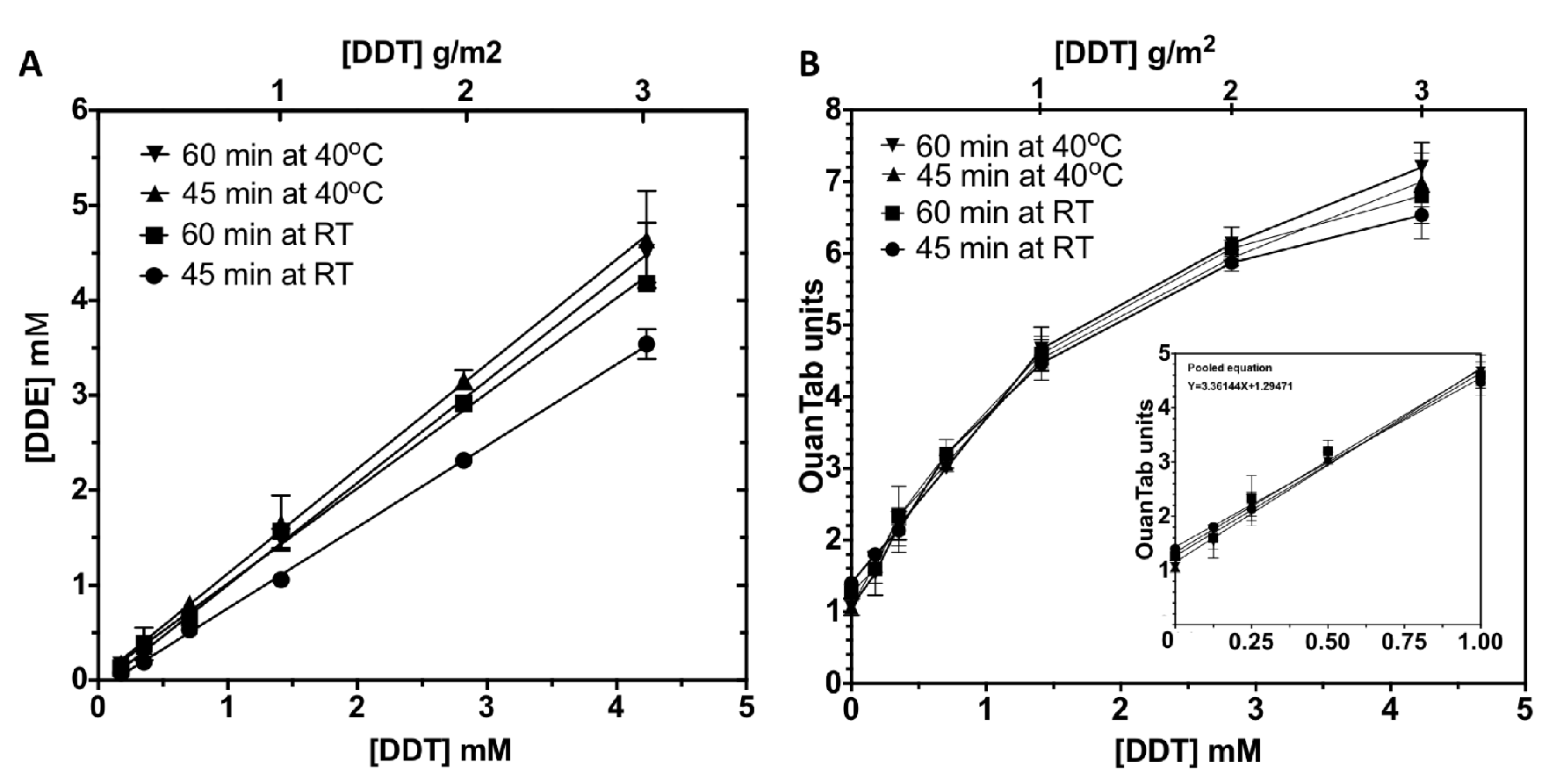

Supplement: S3 Fig — A, DDE production (HPLC measurement), B, Cl- production (Quantab measurement), inset is correlation analysis for the linear portion of the DDT standard curve (0–1 g/m2) the top X-axis shows DDT rates (g DDT/m2) equivalent to the bottom X-axis DDT concentrations (mM). Reactions were carried out at 25°C (RT) and 40°, with 45 and 60 minutes incubation times. (TIFF) [file pntd.0004324.s003.tiff]

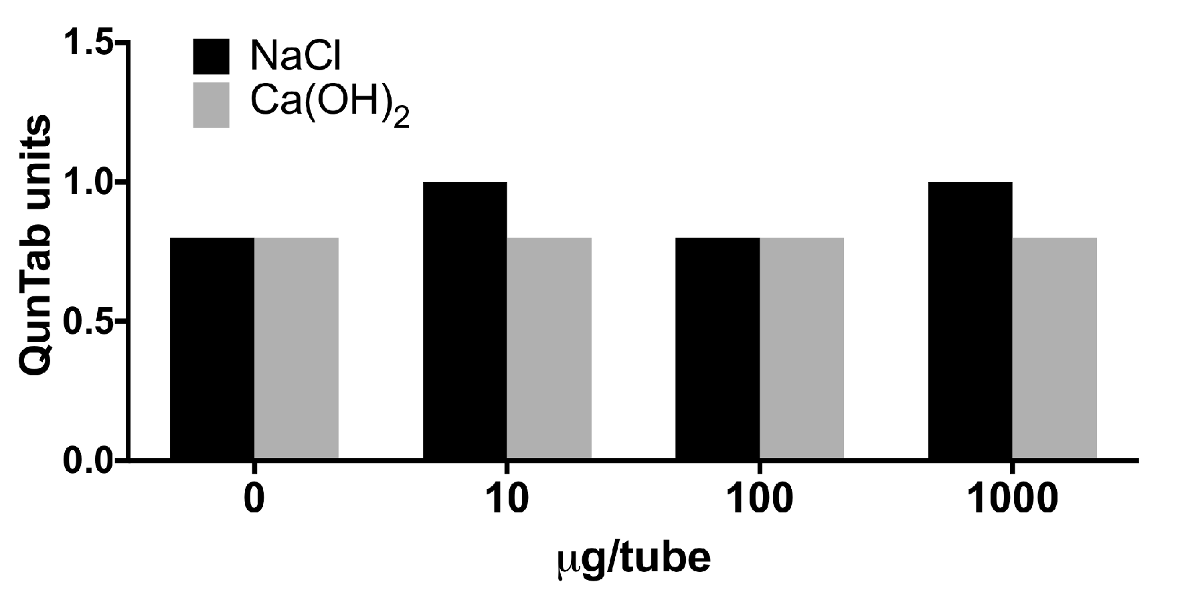

Supplement: S4 Fig — (TIFF) [file pntd.0004324.s004.tiff]

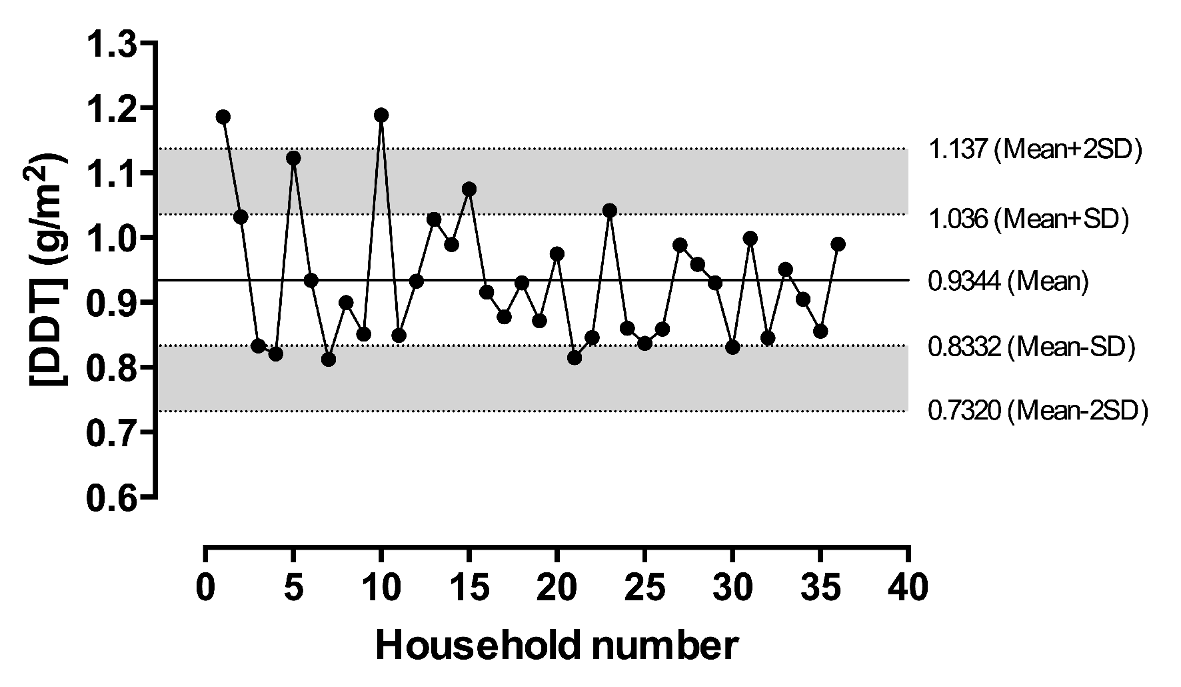

Supplement: S5 Fig — (TIFF) [file pntd.0004324.s005.tiff]
